# How racial‐ethnic, immigration, and 2SLGBTQ+ intersectional self‐efficacy of gender‐sexuality alliance advisors shapes youth members' positive experiences

**DOI:** 10.1111/jora.70243

**Published:** 2026-08-03

**Authors:** Tessa M. L. Kaufman, Wouter J. Kiekens, Maria Di Stasio, Alex Mascarino, Robert A. Marx, V. Paul Poteat

**Affiliations:** ^1^ Department of Pedagogical and Educational Science, Faculty of Social and Behavioural Sciences Utrecht University Utrecht The Netherlands; ^2^ Department of Sociology, Faculty of Behavioural and Social Sciences University of Groningen Groningen The Netherlands; ^3^ Department of Human Services and Early Learning. Faculty of Health & Community Studies MacEwan University Edmonton Alberta Canada; ^4^ Department of Child and Adolescent Development San José State University San José California USA; ^5^ Lynch School of Education and Human Development Boston College Chestnut Hill Massachusetts USA

**Keywords:** gender‐ and sexuality alliances, school experiences, self‐efficacy, sexual orientation and gender identity/experiences, victimization

## Abstract

Gender–sexuality alliances (GSAs) provide critical support for 2SLGBTQ+ youth, yet the role of advisors—particularly in their self‐efficacy to address race‐, ethnicity‐, and immigration‐related issues, including how these intersect with sexual and gender identity, a construct we term racial‐ethnic, immigration, and intersectional self‐efficacy (REISE)—remains understudied. This study examined (a) which advisor‐, GSA‐, and school‐related factors are associated with advisors' REISE, and (b) how this efficacy relates to youth members' socioemotional experiences. Data were collected from 627 GSA members at three time points and from 64 advisors at one time point from 51 GSAs in Massachusetts, New York City, and California. Advisors' REISE was associated with personal, advisor‐related, contextual, GSA‐related, and school‐related factors, and with GSA youth members' reported school experiences. Advisors of color and those who felt more confident in supporting GSA members—particularly regarding 2SLGBTQ+ issues—reported higher REISE. Higher REISE was also observed in advisors of GSAs with a greater proportion of members of color, in schools offering culturally responsive curricula and race/ethnicity‐focused events, and of GSAs characterized by open and inclusive climates. The extent to which advisors felt more efficacious was unrelated to how long they had served as a GSA advisor or to the frequency of GSA meetings. Moreover, random‐intercept multilevel models showed that members with advisors who reported higher REISE perceived their GSAs and advisors to be more supportive at that same time point. Beyond the GSA itself, advisors' higher REISE also predicted higher positive affect and lower victimization among members, with these benefits lasting for several months. These associations did not vary by GSA members' race/ethnicity or victimization, suggesting that advisor REISE may serve as a universal protective resource for diverse youth.

## INTRODUCTION

Gender–sexuality alliances (GSAs) are critical school‐based spaces that promote safety, empowerment, and belonging for youth with diverse sexual orientations and gender identities, including but not limited to youth who are attracted to people with the same or another gender (e.g., gay, lesbian, bisexual) and youth who identify with other gender(s) than their sex at birth—referred to as 2SLGBTQ+ youth (Griffin et al., 2004; Mayberry, 2013; Russell et al., 2009). In the current sociopolitical climate, however, diversity, equity, and inclusion (DEI)‐related efforts, as well as support for 2SLGBTQ+ youth, have become increasingly contested in many school contexts. Although GSAs are not formal DEI programs, they are school‐based spaces in which identity affirmation, inclusion, and dialogue around marginalization are central, and may therefore face similar constraints. Thus, understanding how these spaces can effectively support youth is especially important.

Advisors play a pivotal role in shaping the inclusivity of GSAs. However, many report limited self‐efficacy in supporting racially diverse 2SLGBTQ+ youth (Poteat & Scheer, 2016; Watson et al., 2010). According to Bandura (1997), self‐efficacy refers to individuals' beliefs in their capacity to organize and execute the actions required to manage prospective situations. Building on this foundational concept, we put forward the concept of racial‐ethnic, immigration, and intersectional self‐efficacy (REISE), which reflects advisors' perceived competence in recognizing and addressing racial‐ethnic and immigration marginalization in general and when intersecting with 2SLGBTQ+ identities and marginalization. Such intersection refers to how multiple social identities and systems of marginalization overlap and shape youths' experiences in interconnected ways. When applied to GSA advisors, this construct captures an essential yet understudied aspect of their roles, encompassing their confidence to navigate multiple and overlapping systems of oppression and to foster GSAs that affirm multiple social identities and backgrounds simultaneously. This role is vital because when GSAs explicitly address issues of race, racism, and issues of intersectional oppression, members, regardless of their race/ethnicity, report a stronger sense of belonging and more advocacy (Baams & Russell, 2021; Chong et al., 2019).

Although some work has begun to examine self‐efficacy to address racial and ethnic diversity issues among GSA advisors (Poteat & Scheer, 2016), recent literature reviews recommend research on how this relates to other advisor or school characteristics and how it relates to youths' experiences in GSAs (e.g., Van Vliet et al., 2025). Adopting an intersectional lens is particularly crucial in GSA research, because advisors' work routinely involves supporting young people whose identities and experiences cannot be understood through single social categories alone. An intersectional perspective strengthens our understanding of how advisors can support diverse groups of youth members in ways that are attuned to the interconnected nature of these youths' social positions (Spanierman & Smith, 2017). Addressing this need, the current study examines which advisor, GSA, and school characteristics contribute to advisors' REISE, and how their REISE contributes to positive experiences within and beyond the GSA among youth members.

### Conceptualization of advisors' REISE

Advisors' REISE in the GSA context has been conceptualized as feeling efficacious in addressing issues around race, ethnicity, and immigration in general and at the intersection of 2SLGBTQ+ issues. This includes feeling capable of discussing students' experiences of racism, supporting members around intersectional concerns, understanding immigration‐related and cross‐cultural experiences, taking action to address challenges faced by members of color, and advocating for students whose intersecting identities are marginalized and discriminated against within the broader school context (e.g., Calzo et al., 2021; Poteat et al., 2026). Advisors have named these tasks as central to their role in the GSA (Davis et al., 2024; Valenti & Campbell, 2009).

Advisors' REISE is conceptually related to constructs such as culturally responsive teaching efficacy and intercultural teacher efficacy, which also concern educators' confidence in working with diverse populations. However, REISE differs in two key ways from these two constructs. First, it explicitly centers race‐, ethnicity‐, immigration‐ and related experiences in intersection with sexual and gender identity, rather than diversity more broadly. Second, it is grounded in the GSA context, where advisors are not serving in a didactic instructional role with a clear hierarchy or assessment role over students. Rather, they are often required not only to support youth relationally, but also to facilitate dialogue about marginalization, affirm intersecting identities, and engage in advocacy‐oriented activities with or for youth. As such, REISE reflects advisors' perceived capability to enact these practices in real‐time interactions with youth, rather than broader orientations, knowledge, or attitudes related to diversity.

To explain variability in advisors' REISE and to understand how REISE relates to youth members' experiences over time, we draw on ecological systems theory as a complementary organizing lens. From this perspective, GSAs can be understood as proximal microsystems in which youth interact with advisors and peers, while the broader school environment represents an additional contextual layer that shapes what support is possible and how it is experienced. This broader context is particularly relevant in light of ongoing sociopolitical debates around inclusion, which may influence how GSAs function and the extent to which advisors can enact supportive practices (American Civil Liberties Union, 2025; Kosciw et al., 2022; Watson et al., 2010). Within these nested systems, advisors function as key relational agents whose REISE may shape youth members' experiences. Bandura's account of self‐efficacy development is therefore situated within this broader ecological view, in which advisor‐, GSA‐, and school‐level conditions jointly shape opportunities for efficacy development.

### Explaining variability in advisors' REISE

**FIGURE 1:**
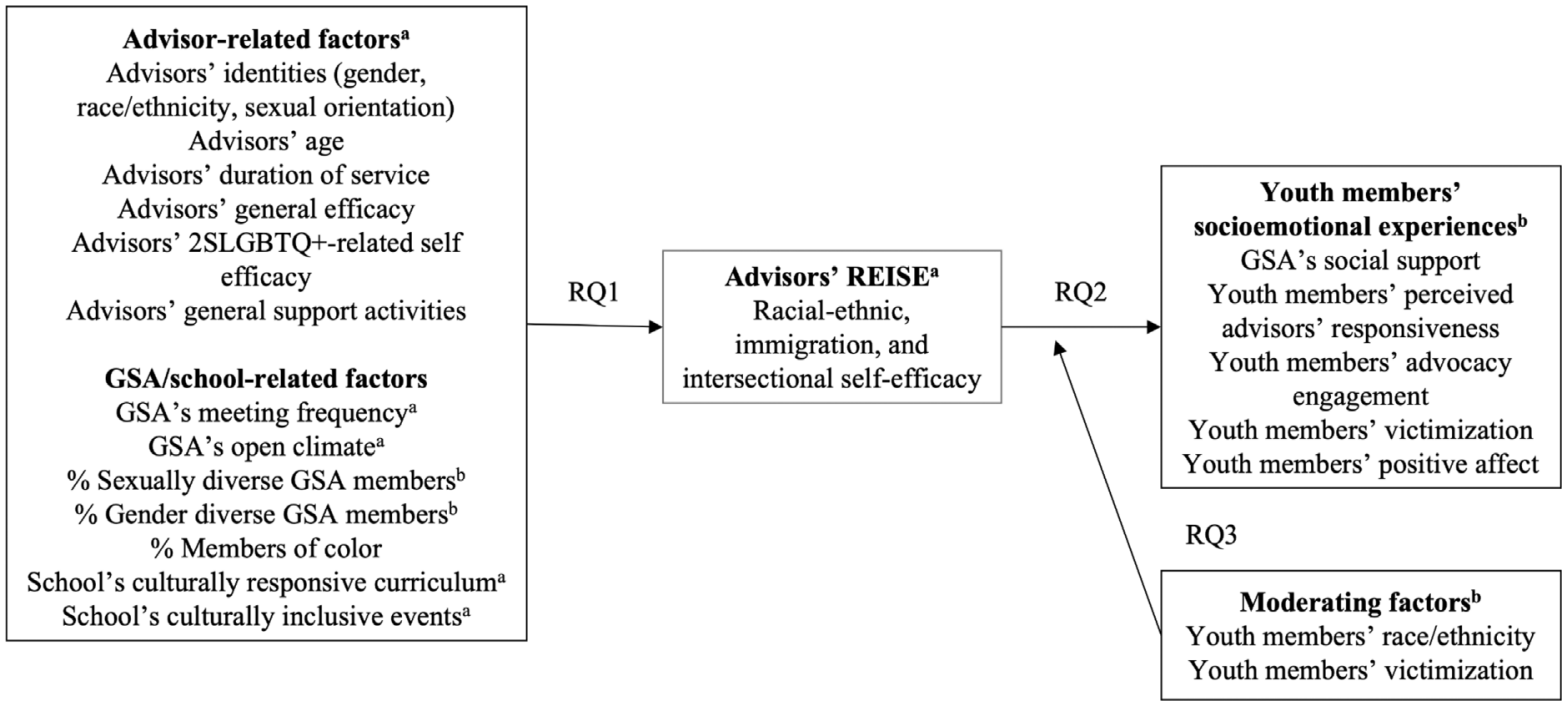
Conceptual model. RQ2‐3 associations were adjusted for GSA meeting frequency, *N* of advisors, and youth members' race/ethnicity, gender, and grade level. ^a^Advisors' reports. ^b^Youth members' reports.

Building on ecological framing, the factors that contribute to advisors' REISE can be understood through a social cognitive lens (Bandura, 1997). According to this framework, self‐efficacy develops through mastery experiences (i.e., successful past experiences), vicarious learning (i.e., learning through observing others), social persuasion (i.e., encouragement or discouragement from others), and affective states (i.e., emotional and psychological reactions). Therefore, REISE is conceptualized not only as a reflection of advisors' backgrounds or contextual conditions, but also as a proximal capacity that is expressed through advisors' actions in supporting youth and addressing intersectional marginalization. As such, REISE may function as a mechanism through which advisor‐, GSA‐, and school‐level conditions contribute to youth experiences. In the GSA context, advisors' REISE is potentially shaped by their personal experiences and practices, as well as by the facilitating factors or barriers present within the GSA or school environment. We consider how each of these domains (advisor‐related vs. GSA‐ and school‐related factors) may relate to advisors' REISE. Figure 1 illustrates the conceptual model, the left part of which focuses on explaining advisors' REISE.

#### Advisor‐related factors

First, advisors may individually differ in their REISE based on their personal experiences. Empirical evidence indicates that advisors with marginalized identities report higher racial and ethnic self‐efficacy (Poteat & Scheer, 2016). Advisors who are racially or ethnically diverse—and possibly also 2SLGBTQ+—may draw upon lived experiences of marginalization. These experiences may function as sources of mastery, such as their own experiences of navigating and intervening when faced with intersectional oppression, or vicarious learning, referring to the successful experiences of people close to them. Such experiences could strengthen their confidence in recognizing and addressing racial‐ethnic marginalization in 2SLGBTQ+ contexts (e.g., Spanierman & Smith, 2017). For example, a GSA advisor who has experienced anti‐Black racism may be more able to identify and offer support when a Black member of their GSA is singled out for exclusionary discipline by school administrators or when a Latine member of their GSA is questioned about their citizenship status.

GSA advisors' age and duration of service as an advisor also may be associated with their REISE. Older advisors or those who have been advisors longer may have had more cumulative opportunities for mastery‐type experiences. Longer‐serving advisors may feel more adept at handling a variety of situations, as they may have previously seen and addressed various issues related to intersectional oppression within their GSAs. On the other hand, newer advisors may have had more recent training, which may function as social persuasion when it involves guidance or feedback, and may additionally provide opportunities for vicarious learning (e.g., observing examples) or mastery experiences (e.g., practicing responses).

Additionally, younger advisors may have come of age and been educated in contexts where diversity and intersectional oppression are more actively discussed (Asher, 2007), and they may therefore feel more comfortable identifying and intervening in situations of oppression. The evidence on the associations between advisor age and duration of service with this form of domain‐specific efficacy is mixed: Poteat and Scheer (2016) found no associations with age or tenure, whereas other work in teacher contexts shows positive links between domain‐specific (i.e., intercultural) efficacy and experience (Romijn et al., 2020). Furthermore, studies indicate that sustained experience with diverse youth and ongoing professional learning contribute to stronger culturally responsive and diversity‐related self‐efficacy (Malinen et al., 2013; Siwatu, 2007).

In addition to advisors' personal background factors, advisors' interactions with students likely play an important role in their REISE. Advisors' REISE could be strengthened by their self‐efficacy in supporting students generally, by their self‐efficacy specific to other topics related to marginalization (i.e., 2SLGBTQ+ related topics), or by greater experience in such types of support (i.e., more frequent general support contributing to more specific REISE). Advisors who provide emotional and social support, foster open dialogue, validate diverse perspectives, and cultivate an open and respectful climate within the GSA in general may engage in more frequent and meaningful advisor–student interactions that function as mastery experiences, such as successfully navigating discussions of personal and sensitive issues related to race, ethnicity, immigration, and their intersection with 2SLGBTQ+ issues. These experiences may be reinforced through positive affective states (i.e., emotional and psychological responses), such as feeling trusted and affirmed by youth, and, when youth provide explicit feedback, through social persuasion (i.e., encouragement from others). These processes may be reciprocal, such that effective support strengthens advisors' self‐efficacy, which in turn further enhances their capacity to address these topics over time (Davis et al., 2024; Lauermann & Berger, 2021). Indeed, empirical studies showed that greater socioemotional competence contributed to GSA advisors' general self‐efficacy (Davis et al., 2022), that teachers' inclusive practices predicted intercultural self‐efficacy (Romijn et al., 2020), and that self‐efficacy in addressing issues for transgender members contributed to GSA advisors' racial and ethnic self‐efficacy (Poteat & Scheer, 2016).

#### GSA‐ and school‐related factors

In addition to advisor‐related factors, Bandura's social cognitive theory (1997) also considers the role that contextual factors can play in creating opportunities for experiences to develop self‐efficacy. In the GSA context, for example, advisors of GSAs with more members of color may have more opportunities for conversations and interactions with these youth, which may provide additional mastery experiences and opportunities for vicarious learning. For example, they may learn about their unique experiences and serve as a stronger ally in providing support and facilitating conversations and advocacy in the GSA around issues of racial discrimination (Calzo et al., 2020; Poteat & Scheer, 2016; Singh et al., 2024). Similarly, advisors of GSAs with more 2SLGBTQ+ members may have more opportunities for understanding the experiences of marginalized youth, and they may be able to apply that knowledge and empathy to issues of other forms of oppression and intersectional oppression. Likewise, regardless of member composition, a GSA with a more open climate for respectful discussion likely could facilitate advisors' REISE by supporting positive affective states (e.g., feeling safe and confident engaging in these discussions); such a climate has also been shown to relate to moderately higher levels of advisors' 2SLGBTQ+‐related self‐efficacy (O'Brien et al., 2023). Lastly, GSAs that meet more frequently could provide advisors with more experience relevant to handling diverse topics than those that might meet less often and therefore may have fewer conversations around various forms of oppression.

Regarding the broader school environment, school‐level features such as inclusive curricula, equity‐oriented policies, and a supportive climate may strengthen advisors' perceptions that addressing racial, ethnic, immigration, and intersectional oppression is a legitimate and necessary part of their role. Such processes likely occur by creating opportunities for mastery experiences, for example by providing the space and support to address these issues in practice, and by fostering positive affective states, such as feeling supported and safe in doing so. For example, a GSA advisor who works in a school that fails to act on race‐ or ethnicity‐based bullying may feel less efficacious in their abilities to foster an inclusive GSA climate for members of color. Consistent with this idea, GSA advisors view school policies and school‐based resources as crucial to carrying out their roles (Watson et al., 2010), and evidence across 46 countries indicates that inclusive school climates are associated with higher teacher intercultural self‐efficacy (Schwarzenthal et al., 2023).

### Advisors' REISE and youth members' socioemotional experiences

In addition to explaining variability in advisors' REISE, it is vital to understand how REISE relates to youth members' positive socioemotional experiences—both within and beyond the GSA (Figure 1, right portion). The positive youth development (PYD) framework posits that supportive adults and environments play a central role in fostering youth resilience, belonging, and well‐being (Lerner et al., 2015). These processes are especially salient during adolescence, a developmental period characterized by identity formation, social belonging, and socioemotional regulation. Within GSAs, advisors may therefore function as important non‐parental developmental assets, as PYD frameworks emphasize the role of supportive adult relationships in fostering youth development (Lerner et al., 2009). In line with this, research shows that advisor characteristics and involvement are associated with youth well‐being outcomes such as purpose, mastery, and self‐esteem within GSAs (Poteat et al., 2016). Advisors may contribute by shaping group processes, facilitating supportive relationships, and enabling youth leadership and engagement, which are central mechanisms through which GSAs promote positive development. From this developmental lens, advisors' REISE may matter because it shapes how effectively advisors can respond to identity‐related challenges in ways that support belonging, emotional security, and well‐being.

Accordingly, advisors with greater REISE may be more able to support 2SLGBTQ+ youth in GSAs who more often face invisibility or experience unique stressors due to intersectional oppression. These advisors may thus be in a better position to promote all their members' empowerment and well‐being. Indeed, studies show that teachers' intersectional competence predicted more supportive and psychologically safe school climates (Bayram Özdemir et al., 2024), and that teachers' culturally relevant teaching practices are associated with higher levels of students' school belonging (Byrd, 2016).

Beyond the specific GSA context, youth members who receive support in navigating issues related to their intersectional identities—for example, in coping with stigma—likely feel generally happier and more emotionally secure, consistent with research linking identity‐affirming adult support to greater well‐being and positive affect among marginalized youth (McGuire et al., 2010). Moreover, these youth likely experience less bullying‐victimization; GSA presence has been shown to predict decreased levels of bullying‐victimization, particularly among students of color (Van Vliet et al., 2025). Advisors' REISE may play a role in this by their ability to discuss solutions or feeling empowered to challenge mistreatment. Indeed, teacher support plays a key role in mitigating identity‐based bullying (Sainju et al., 2025), and teachers with greater self‐efficacy and more affirming attitudes toward marginalized youth are also more likely to intervene in discriminatory bullying and create safer peer climates (Collier et al., 2015).

### Differential associations of advisor REISE across youth subgroups

Because REISE pertains to advisors' confidence in addressing racialized dynamics within 2SLGBTQ+ contexts, it may be relevant for all youth members, as such competencies can contribute to more inclusive, affirming, and responsive group environments overall. Advisors with higher REISE may be better equipped to recognize exclusionary dynamics as they emerge, foster a sense of belonging among group members, and respond to diverse needs in thoughtful and context‐sensitive ways. This may include being more attuned to how race and ethnicity intersect with other aspects of identity, more likely to address tensions or inequities within the group arising from racism and extending to reasons other than racism, and more capable of facilitating discussions or interactions that affirm multiple perspectives. Through these processes, advisors' REISE may contribute to group climates that feel supportive, inclusive, and validating for a wide range of youth.

However, while holding potential benefits to all youth, the associations between REISE and positive member experiences may be even more pronounced for certain subgroups. Given that advisors' REISE pertains most closely to the lived experiences of youth members of color, these members might reap stronger benefits than their White peers because they feel more validated and supported along this dimension of their identity. These differential benefits may follow a similar pattern to what has been observed with GSA advisors' self‐efficacy to address transgender issues, which was related to reduced depressive symptoms only among transgender members, and not their cisgender peers (Andrzejewski et al., 2023). Similarly, victimization may moderate the association between advisor REISE and youth outcomes. Advisor REISE may be more strongly associated with better well‐being among youth who experience higher levels of victimization, because these youth are more likely to need identity‐sensitive responses to handle stressors. Overall, we investigate whether the potential impact of advisors' REISE is stronger for youth members of color than for White members, and for members with higher victimization levels.

### Current study

Although GSA advisors clearly hold the potential to promote youth members' positive socioemotional experiences, research is needed on the factors that are tied to advisors' REISE and how REISE may relate to youth members' experiences over time (e.g., Poteat et al., 2017; Van Vliet et al., 2025). Such insights could inform efforts aimed at strengthening advisors' ability to support youth from these diverse backgrounds in a way that is attuned to multiple and intersectional forms of oppression. Guided by an ecological systems perspective, and drawing on social cognitive and PYD frameworks, the present study examines how advisor‐, GSA‐, and school‐level factors related to the development of REISE and its implications for youth outcomes.

By centering advisor processes within diverse and racially mixed GSA contexts, the current study aimed to clarify how advisors' REISE may function as a resource—consistent with social cognitive theory—through which GSAs foster inclusion, reduce victimization, and strengthen well‐being for youth experiencing oppression. As shown in Figure 1, our first research question (RQ1) examined how REISE can be explained by individual advisor factors (advisor background: specifically identities, age, and duration of service, support indicators) and contextual factors related to the GSA (i.e., member composition in terms of diversity, meeting frequency), and related to the school (i.e., the school's culturally inclusive curricula and events), reflecting multiple levels of youths' social ecologies. Our second research question (RQ2) examined whether advisors' REISE predicted youth members' school experiences, including GSA‐related experiences (perceived advisor responsiveness, GSA social support, advocacy engagement) and general experiences beyond the GSA (victimization, positive affect). Consistent with a PYD perspective, these outcomes reflect key indicators of adolescents' socioemotional functioning and well‐being. Our third research question (RQ3) examined whether the associations between advisors' REISE and youth members' experiences were stronger for members of color than for White members, and for those with higher victimization levels. We addressed our questions within a multi‐informant, longitudinal framework to capture both GSA advisor and youth members' experiences, across advisors' and youths' individual and contextual school/GSA levels.

## METHODS

Participants were 64 advisors and 627 youth members from 51 GSAs in Massachusetts (*n* = 319), New York City (*n* = 103), and Northern and Southern California (*n* = 205), recruited through our professional networks with attention to diversity in terms of school and GSA size, racial‐ethnic diversity, and urbanicity. Although GSAs were all recruited from politically progressive states, in each state we sought to recruit GSAs in areas that were relatively more politically conservative based on both our informal conversations with professional contacts at these schools and past voting histories in the locality or localities served by the schools. The schools also ranged from predominantly White students to predominantly students of color. Schools included traditional public schools as well as vocational‐technical public schools (e.g., those in which students learn professional trades in addition to the standard curricula).

GSA advisors who expressed interest in the project secured permission from their school principal for us to work with their GSA. We visited GSAs in person or virtually during a GSA meeting to describe the project to youth members. Consistent with the university IRB regulations and best practices for conducting research with marginalized participants who may be adversely impacted by obtaining parental consent (Sims & Nolen, 2021), GSA advisors provided consent for their own participation, and they also provided adult consent—in place of parental consent—for youth members. Members also assented to participate. In two study sites, IRB regulations required youth to obtain parental consent; the consent form at these study sites did not mention GSAs and instead referred to extracurricular clubs. After the consent process, GSA members and advisors completed their first survey in the same time window. GSA members later completed two more surveys, each spaced 2–3 months apart; of the 627 youth who participated at T1, *N* = 300 participated at T2 and *N* = 201 at T3. Data collection occurred during the 2021–2022 and 2022–2023 school years. Advisors and youth members received a $10 online gift card to compensate them for completing each 20–30 min survey. All procedures were approved by the Institutional Review Board of Boston College (Protocol 19.280.01). We explored potential patterns of missingness in our data. A total of 135 youth (21.5%) completed all three surveys, whereas 127 youth (20.3%) completed two surveys, and 365 youth (58.2%) completed one survey. With small correlations, youth who participated in more waves perceived relatively higher levels of responsiveness from their advisor at wave 1 (*r* = .14, *p* = 003) and reported more involvement in advocacy for marginalized people beyond the 2SLGBTQ+ community (*r* = .25, *p* < .001), but this was not correlated with their age (*r* = .03, *p* = .49), or wave 1 perceived social support in the GSA (*r* = .03, *p* = .55), victimization (*r* = −.01, *p* = .87), or positive affect (*r* = .07, *p* = .12). Participation rates did not differ by sexual orientation (*F* (1, 591) = 0.006, *p* = .94), race/ethnicity (*F* (1, 579) = 0.025, *p* = .88), or gender identity (*F* (2, 510) = 0.661, *p =* .52). Additionally, a random‐intercept two‐level model showed that participation rates were not associated with advisors' REISE (*b* = −.06, *p* = .29).

### Measures

#### Advisors reports

##### Advisors' background

Advisors reported on their background, including age, identities (gender identity, race/ethnicity, sexual orientation), and tenure as a GSA advisor. To measure gender identity, we asked advisors to check all options that they would use to identify their gender identity, with the options *male*, *female*, *questioning*, *non‐binary*, *genderqueer*, *genderfluid*, *agender*, write‐in, or *prefer not to answer*. We also asked advisors for their sex assigned at birth, with the options *male, female, intersex*, and *prefer not to answer*. This information was recoded by the research team into 14 categorical options (“cis male,” “cis female,” “questioning,” “non‐binary,” “gender queer,” “gender fluid,” “agender,” “bigender,” “masculine gender expansive,” “feminine gender expansive,” “gender expansive,” “trans [no additional information provided],” “trans male,” “trans female”). For the purposes of this analysis, these 14 categories were then dichotomized into cisgender participants (those who indicated a cis male or cis female identity) and gender diverse participants (those who indicated any other gender identity). Additionally, the cisgender categories were dichotomized into cisgender male and cisgender female.

The race/ethnicity item included eight categorical response options, with the possibility to check all that applied (*White/European American*, *Black or African American*, *Asian/Asian American*, *Latino/a/x*, *Bi/Multiracial*, *Native American*, *Middle Eastern/Arab or Arab‐American*, and write‐in). For the purposes of this analysis, responses were dichotomized into White (those who indicated only a White race/ethnicity) and Advisors of Color (those who indicated any other racial‐ethnic identity).

The sexual orientation item included eight options, with the possibility to check all that applied (*gay or lesbian*, *bisexual*, *questioning*, *heterosexual/straight*, *pansexual*, *asexual*, *queer*, and write‐in). For the purposes of this analysis, we dichotomized responses into straight (those who indicated they were heterosexual or straight) and sexually diverse (those who indicated any other identity). Finally, advisors reported their duration of service as GSA advisors, expressed in months.

##### Advisors' REISE, general self‐efficacy, 2SLGBTQ+‐related self‐efficacy

###### Advisors' REISE

Advisors reported on their perceived REISE, using a scale developed by the research team over the last 10 years and based on their experiences working with GSAs and advisors (e.g., Poteat & Scheer, 2016). The scale included eight items on how competent advisors felt in supporting youth members of color and immigrant youth, and issues faced by youth who experienced minoritization along multiple axes related to race, immigration, sexual orientation, or gender identity. A complete list of items is presented in Supplementary Material A, Table A1. Answer options ranged from 1 (not at all) to 5 (extremely), and items were averaged to compute composite scores. We conducted an exploratory factor analysis with the eight items using maximum likelihood extraction and retaining factors with eigenvalues greater than 1.0. The results supported a unidimensional factor structure, which had an eigenvalue of 6.34 (the eigenvalue dropped to 0.59 for the potential second factor) and accounted for 76% of the variance. Factor loadings ranged from .82 to .93. The scale showed excellent internal reliability (*α* = .96).

##### Advisors' general self‐efficacy

Advisors reported their perceived general self‐efficacy in providing support to youth members through four items. Respondents were asked how competent they felt supporting youth members during or outside of a GSA meeting, across different domains regarding: struggles at home, academically, in peer relationships (i.e., experiencing bullying/discrimination), and school absenteeism. A complete list of items is presented in Supplementary Material A, Table A2. We generated these items based on prior research documenting that these are among the common issues for which GSA advisors provide support to youth members (Davis et al., 2024; Valenti & Campbell, 2009). Response options ranged from 1 (not at all) to 5 (extremely), and items were averaged to compute composite scores. The scale showed good internal reliability (*α* = .80).

##### Advisors' 2SLGBTQ+‐related self‐efficacy

Advisors also indicated their perceived 2SLGBTQ+‐related self‐efficacy, which included 14 items (7 items related to sexual diversity and 7 items related to gender diversity; see Supplementary Material A, Tables A3 and A4) developed by the research team over the last 10 years (e.g., Poteat & Scheer, 2016). For example, advisors indicated how competent they currently felt to talk about unique experiences that transgender and non‐binary students face, and that LGBQ students face. Answer options ranged from 1 (not at all) to 5 (extremely) and items were averaged to compute composite scores. The scale showed excellent internal reliability (*α* = .93).

##### Advisors' general support activities

Additionally, advisors reported on nine items assessing how often they *engaged* in different support activities during or outside of a GSA meeting, across different domains (e.g., “*Met individually with a student in the GSA who was going through a difficult time*”). We generated these items to reflect common supportive efforts provided by GSA advisors and by school staff in general to students (Davis et al., 2024; Tennant et al., 2015; Valenti & Campbell, 2009). Answer options ranged from never to 5+ times on a 5‐point scale, and items were averaged to compute composite scores. The scale showed good internal reliability (*α* = .87).

##### GSA's open climate

Advisors rated the extent to which their GSA fostered an open and participatory environment on four items (e.g., “*Students can disagree with the advisor if they are respectful*”), adapted from the Open Classroom Climate Scale (Flanagan et al., 2007). Answer options ranged from 1 (strongly disagree) to 5 (strongly agree), and items were averaged to compute composite scores. The scale showed good internal reliability (*α* = .82).

##### GSA's meeting frequency

Advisors reported on how often their GSA had met so far during the school year, with response options ranging from 1 (no regular meetings scheduled) to 6 (more than once a week).

##### School's culturally inclusive curriculum and events

Advisors indicated at what stage their school's policies and procedures were in terms of (1) a “*culturally relevant and responsive curriculum that reflects all students throughout the school year (not just special days or months)*” and (2) “*attention to experiences related to race (e.g., Black history month)*.” The response options and relative descriptions provided were: *beginning* (initial stages of change; very little to no implementation; limited or vague understanding of the issues); *exploring* (beginning stages of implementation and change; early stages of understanding student needs and benefits of change); *developing* (intermediate stages of implementation and change; moderate understanding of student needs and benefits of change); and *mastering* (well developed and comprehensive implementation; thorough and complex understanding of needs and benefits of change).

#### Youth members reports

##### Youth members' background

Youth members reported on their age, identities (gender diversity, race/ethnicity, and sexual orientation) using similar measures and classifications as advisors.

##### GSA's social support

Youth members reported on five items from the GSA Involvement Scale (Poteat et al., 2016) asking about the role of GSAs as a place of support (e.g., “*So far this year, for me, my GSA is a place where I receive validation and reassurance*”). Response options ranged from 0 (never) to 4 (very often), and items were averaged to compute composite scores. Youth members also had the option “prefer not to answer,” which was coded as missing. The scale proved good internal consistency across all time points (*α* ^T1^ = .80; *α* ^T2^ = .84; *α* ^T3^ = .90).

##### Perceived advisors' responsiveness

Youth members also indicated how they felt about their advisors' responsiveness to their needs (e.g., “*I like the way my GSA advisor treats me when I need help*”) using the 7‐Item Care subscale from the Tripod study (Ferguson & Danielson, 2014). Response options ranged from 1 (totally untrue) to 5 (totally true), and items were averaged to compute composite scores. Youth members also had the option “Prefer not to answer,” which was coded as missing. Internal consistency was excellent across all time points (*α* ^T1^ = .90; *α* ^T2^ = .90; *α* ^T3^ = .93).

##### Youth members' advocacy engagement

The extent to which youth members were involved in advocacy activities for marginalized people beyond the 2SLGBTQ+ community in their GSA was measured through one item (e.g., “*Activities for marginalized groups beyond the LGBTQ community, like for race, immigration, or ability*”) with response options ranging from 0 (Not at all involved) to 4 (Extremely involved). This item was only completed by members who reported that they received opportunities for such engagement, and those without opportunities were not included in these analyses to distinguish opportunities from engagement.

##### Youth members' victimization

Youth members reported on three items assessing how often they experienced different types of victimization in the past 30 days in or out of school: (1) being hit or pushed by other students, (2) being picked on or made fun of, and (3) being excluded from conversations or activities. Response options ranged from 0 (0 times) to 4 (7+ times). These items capture the three central forms of bullying commonly distinguished in the literature, namely physical, verbal, and relational victimization and are consistent with widely used measures of peer victimization (Modecki et al., 2014). In line with prior research conceptualizing peer victimization as a multifaceted construct reflected across these forms, and given that, advisors' REISE is not specific to any particular form of victimization but reflects general practices relevant across forms, items were combined into a composite score. The scale showed questionable to acceptable internal consistency (*α* ^T1^ = .68; *α* ^T2^ = .70; *α* ^T3^ = .59); McDonald's omega (*ω* ^T1^ = .72, *ω* ^T2^ = .72, *ω* ^T3^ = .61), which may partly reflect the small number of items and the multidimensional nature of victimization. However, mean inter‐item correlations were moderate to high for every time point (*r* = .31–.54), further supporting that the items function as a composite.

##### Youth members' positive affect

Finally, youth members reported on their positive affect, measured through the I‐PANAS‐SF (Thompson, 2007), which includes a 5‐item subscale for positive affect specifically. Items included feeling alert, inspired, determined, attentive, and active. Response options ranged from 1 (very slightly/not at all) to 5 (extremely), and items were averaged to compute composite scores. The scale showed questionable to acceptable internal reliability across time points (*α* ^T1^ = .67; *α* ^T2^ = .64; *α* ^T3^ = .70); inter‐item correlations ranged between *r* = .09 and *r* = .45, and none of the items reduced the measure's reliability.

### Analytic plan

All analyses were conducted in Mplus 8.11 using the MLR estimator, which accounts for non‐normality and handles missing data through full information maximum likelihood (FIML). To address RQ1 (identifying advisor and contextual factors concurrently associated with advisors' REISE), individual advisor‐level correlations were computed between individual advisors' REISE and their background characteristics (i.e., advisors' ethnic/racial and sexual/gender identities, age, duration of advising) and support indicators (i.e., advisors' perceived general and 2SLGBTQ+‐related self‐efficacy, and their actual support activities).

Second, we examined school/GSA‐level associations between advisors' REISE and GSA or school characteristics. We estimated two‐level models to account for the nesting of individual youth member reports within schools/GSAs and to separate individual member‐level variability from school/GSA‐level variability. At the between level, we estimated correlations between advisors' REISE and school/GSA‐level indicators of GSA membership composition, GSA open climate, and culturally inclusive school practices. Membership composition included racial‐ethnic and sexual/gender diversity indicators based on member reports. GSA open climate, inclusive curriculum, and inclusive events were based on advisor reports. Adjustment for the number of advisors per GSA was implemented within the two‐level model by regressing both advisors' REISE and the school/GSA‐level indicators on the number of advisors at the between level. The resulting associations therefore represent residual, or partial, between‐level correlations. Results were comparable when this adjustment was omitted.

Further, RQ2 tested whether advisors' REISE was associated with youth members' socioemotional experiences at all time points (T1–T3) again using two‐level random‐intercept models. We estimated associations between advisors' REISE and youth members' experiences at the school/GSA level, while also adjusting for GSA meeting frequency and number of advisors, the school's culturally inclusive curriculum and events, and youth members' individual race/ethnicity, sex at birth, gender, and grade level. Cross‐sectional (T1) associations were estimated in a single multilevel model including all outcomes simultaneously, whereas longitudinal (T2 and T3, adjusted for baseline levels of the outcomes) estimates of the same associations were tested in separate models for GSA‐based versus general experiences to reduce model complexity.

Lastly, RQ3 examined whether the link between advisors' REISE and GSA member experiences differed for members of color relative to White members and across victimization levels. Cross‐level interaction effects estimated whether associations between Level‐1 GSA member race/ethnicity and victimization at T1 and GSA member experiences differed across levels of Level‐2 advisors' REISE.

## RESULTS

### Explaining advisors' REISE by advisor‐, GSA‐, and school‐related factors

Descriptive statistics were computed for GSA composition and youth members' outcomes (see Table 1; for all correlations between REISE and youth members' outcomes, see Supplementary Material B). The first research question examined which advisor‐, GSA‐, and school‐related factors were associated with advisors' REISE. For advisor factors, advisors of color reported significantly higher REISE compared to White advisors, *F*(1, 61) = 4.75, *p* = .033, *η* ^*2*^ = .07. No effects emerged along the lines of sexual orientation, *F*(1, 52) = 1.11, *p* = .297, *η* ^*2*^ = .02, or gender identity, *F* (2, 510) = 0.661, *p =* .52. There were no significant correlations (Table 2) between advisors' REISE and advisors' age, nor for their duration of service as an advisor. However, advisors reported substantially greater REISE when they felt more efficacious in supporting youth members generally, particularly regarding 2SLGBTQ+ topics.

**TABLE 1:** Descriptive statistics for predictors and outcomes based on advisors' REISE.

	*M* (SD) or %	Range
Advisors' REISE	3.62 (1.01)	1.00–5.00
*Independent variables (RQ1)*		
Advisor‐related factors		
Age	43.97 (11.15)	21–64
Race/ethnicity (of color)	25%	
Sexual orientation (diverse)	53%	
Gender (diverse)	9%	
Duration of service (months)	63.51 (62.99)	2–360
General self‐efficacy	2.93 (0.83)	1.00–5.00
2SLGBTQ‐related self‐efficacy	3.76 (0.64)	1.57–5.00
General support activities	1.64 (1.08)	0.00–5.00
GSA‐related factors		
Youth members' age (covariate)	15.13 (1.34)	11–22
% Youth members of color	47%	
% Youth members with sexually diverse orientation	92%	
% Youth members with diverse gender identity	54%	
GSA's meeting frequency	4.75 (0.66)	2.00–6.00
GSA's open climate	4.61 (0.55)	3.00–5.00
School‐related factors		
School's culturally inclusive curriculum	2.43 (0.90)	1.00–4.00
School's culturally inclusive events	2.79 (0.87)	1.00–4.00
*Dependent variables (RQ2‐3)*		
Perceived advisors' responsiveness		
Time 1	4.40 (0.66)	1.29–5.00
Time 2	4.43 (0.63)	2.43–5.00
Time 3	4.31 (0.79)	1.00–5.00
GSA's social support		
Time 1	4.14 (0.67)	2.00–5.00
Time 2	4.25 (0.66)	1.60–5.00
Time 3	4.11 (0.84)	1.00–5.00
Advocacy engagement		
Time 1	1.61 (1.38)	0.00–4.00
Time 2	1.89 (1.26)	0.00–4.00
Time 3	1.78 (1.44)	0.00–4.00
Victimization		
Time 1	1.54 (2.22)	0.00–12.00
Time 2	1.19 (2.01)	0.00–12.00
Time 3	1.53 (2.09)	0.00–9.00
Positive affect		
Time 1	2.94 (0.83)	1.00–5.00
Time 2	2.93 (0.78)	1.00–5.00
Time 3	3.00 (0.83)	1.00–5.00

**TABLE 2:** Advisor‐level correlations with advisors' REISE, *N* = 64.

Variable	1	2	3	4	5	6
1. Advisors' REISE	—	−.04	−.02	.36**	.62**	.30*
2. Advisors' age		—	.32	.08	−.11	−.25
3. Advisors' duration of service			—	.01	.14	−.01
4. Advisors' general self‐efficacy				—	.68**	.30*
5. Advisors' 2SLGBTQ+‐based self‐efficacy					—	.33**
6. Advisors' general support activities						—

*Note*: Entries above the diagonal.

* *p* < .05;

** *p* < .01.

Broader GSA‐ and school factors were also associated with advisors' REISE (Table 3). There was a moderate and significant positive association between advisors' REISE scores and the percentage of students of color in a GSA. Advisors' REISE was not significantly associated with the percentage of students with diverse sexual orientations, but showed a moderate, non‐significant negative association with the percentage of students with diverse gender identities. In addition, advisors' REISE was moderately and significantly associated with a more open GSA climate, as well as with the inclusiveness of the school's curriculum and cultural events. There was no significant association between advisors' REISE scores and the frequency of GSA meetings.

**TABLE 3:** School/GSA‐level partial correlations with advisors' REISE (adjusted for N advisors within a GSA), *N* = 51.

Variable	1	2	3	4	5	6	7	8
1. Advisors' REISE	—	.40**	−.12	−0.43	.31*	−.16	.30*	.47**
2. % Youth members of color		—	−.57**	−.37**	.11	.31*	.15	.38**
3. % Youth members with diverse sexual orientation			—	.61**	−.06	−.21*	−.25*	−.37*
4. % Youth members with diverse gender identity				—	.15	−.36*	−.20	−.35*
5. GSA's open climate					—	.20	.03	.13
6. GSA's meeting frequency						—	−.12	−.13
7. School's culturally inclusive curriculum							—	.69***
8. School's culturally inclusive events								—

*Note*: Values represent results from two‐level models estimating GSA‐level partial correlations adjusted for the number of advisors within each GSA (Level‐2) and individual member variability (Level‐1). Two‐tailed tests.

* *p* < .05;

** *p* < .01;

*** *p* < .001.

### Associations between advisors' REISE and youth members' socioemotional experiences

Next, two‐level random‐intercept models showed that advisors' REISE was associated with GSA members' experiences (Table 4). In GSAs where advisors reported, on average, greater REISE, members perceived their GSA to be significantly more supportive and they perceived their advisors as significantly more responsive to their needs. These associations were only observed cross‐sectionally at Time 1 and were not observed with the outcomes as measured at Time 2 or Time 3. Notably, advisors' REISE was not significantly related to youth members' engagement in advocacy activities around intersectionality experiences—although this finding should be interpreted with caution because this analysis only included half of the sample, as half of the GSAs did not provide opportunities to engage in any advocacy activities, preventing them from engaging in such activities.

**TABLE 4:** Estimates from two‐level random‐intercept models of advisors' REISE predicting youth members' school experiences.

Youth members' experiences	T1				T2				T3			
	*b*	95% CI	*β*	ICC	*b*	95% CI	*β*	ICC	*b*	95% CI	*β*	ICC
GSA‐related												
GSA's social support	.09*	0.02; 0.16	.55	.07	−.01	−0.07; 0.06	−.10	.06	−.02	−0.14; 0.10	−.07	.17
Perceived advisors' responsiveness	.10***	0.04; 0.17	.61	.11	.02	−0.09; 0.14	.19	.09	.05	−0.09; 0.20	.34	.13
Advocacy engagement	.06	−0.16; 0.28	.14	.15	.16	−0.07; 0.39	.63	.10	−.02	−0.31; 0.35	−.07	.08
General												
Victimization	−.32*	−0.62; −0.02	−.49	.15	−.20	−0.42; 0.02	−.36	.14	−.27*	−0.52; −0.03	−.58	.18
Positive affect	.10*	0.01; 0.18	.63	.07	.09*	0.00; 0.19	.62	.08	−.01	−0.13; 0.10	−.07	.16

*Note*: Analyses controlled for individual (Level‐1) variability in youth members' race/ethnicity, sex at birth, gender diversity, grade, GSA meeting frequency, the school's culturally inclusive curriculum and events, and (for T2‐T3 models) baseline estimates of the outcomes.

* *p* < .05;

*** *p* < .001.

Further, advisors' REISE was associated with member experiences beyond the GSA. Higher advisors' REISE was associated with lower victimization levels cross‐sectionally and showed a non‐significant, moderately large reduction in victimization after 2 to 3 months (*p* = .099) which strengthened in the following months, resulting in a significant and substantial decrease in victimization (T3). For positive affect, advisors' REISE was associated with higher levels of youth members' positive affect both cross‐sectionally and several months later (T2). Altogether, advisors' REISE was associated with youth members' concurrent GSA experiences, as well as more positive general socioemotional experiences that members reported later in the school year.

### Cross‐level interactions with youth members' race/ethnicity and victimization

Lastly, we tested whether the associations between advisor REISE and youth members' experiences were stronger among members of color or members who were more victimized. The cross‐level interaction estimates were small and statistically non‐significant across outcomes; specifically, for the *GSA's social support* (race/ethnicity: *b* = 0.05, 95% CI [−0.16, 0.28]; victimization: *b* = 0.01, 95% CI [−0.03, 0.45]), *perceived advisor responsiveness* (race/ethnicity: *b* = 0.09, 95% CI [−0.12, 0.29]; victimization: *b* = −0.02, 95% CI [−0.05, 0.02]), *advocacy engagement* (race/ethnicity: *b* = 0.02, 95% CI [−0.61, 0.76]; victimization: *b* = −0.69, 95% CI [−0.17, 0.042]), *victimization* (race/ethnicity: *b* = 0.43, 95% CI [−0.21, 1.13]), and *positive affect* (race/ethnicity: *b* = −0.01, 95% CI [−0.30, 0.25]; victimization: *b* = −0.01, 95% CI [−0.05, 0.03]). Altogether, concurrent associations between advisor REISE and youth members' outcomes were not moderated by youth members' race/ethnicity or victimization levels. Given the modest precision of these estimates based on their confidence intervals and sample size, particularly for some outcomes and interactions with race/ethnicity, these null interaction findings should be interpreted cautiously and as exploratory. Although the sample was large overall, the study was likely better powered to detect main effects than cross‐level interaction effects.

## DISCUSSION

Although GSA advisors are thought to play an important role in promoting members' positive socioemotional experiences, little is known about the factors associated with advisors' racial‐ethnic, immigration, and intersectional self‐efficacy (REISE) and how REISE relates to youth members' experiences over time. Addressing this gap, our findings indicate that GSA advisors' REISE is shaped by both individual and contextual factors and is meaningfully associated with youth members' socioemotional experiences, both concurrently and, beyond the GSA context, across the school year. Importantly, advisors' REISE was also associated with more positive socioemotional experiences among youth members. Such effects were consistent across youth members, regardless of their race/ethnicity or prior levels of victimization, suggesting that REISE may be associated with positive socioemotional experiences across diverse youth populations. The findings support a Positive Youth Development (PYD) framework in which supportive relationships with non‐parental adults function as key developmental assets that promote adolescents' well‐being. Advisors with higher REISE may be better equipped to provide the relationship support, validation, and guidance that foster belonging and emotional security among youth. From an ecological perspective, these effects likely extend beyond the GSA and relate to broader school experiences such as victimization and well‐being.

### Advisor and contextual correlates of advisors' REISE

When explaining advisors' REISE by advisor‐related factors, advisors' racial identity and their self‐reported confidence in supporting youth members, particularly around 2SLGBTQ+ issues, were more strongly associated with REISE than traditional experience‐based indicators (e.g., advisors' age, the number of years served, or how often the GSA met). Higher REISE was also reported by advisors working in more diverse and supportive GSA and school contexts, including GSAs with a greater proportion of members of color, more open and inclusive climates, and schools offering culturally responsive curricula and race‐ or ethnicity‐focused events. REISE may therefore be a form of self‐efficacy more closely tied to identity‐ and context‐specific experiences than to general role duration or structural involvement. This aligns with self‐efficacy theory (Bandura, 1997), suggesting that individuals build perceived capability through mastery experiences, social modeling, and affirmation—not merely through exposure or repetition. Advisors may feel more efficacious when they have encountered situations that required them to navigate identity‐related challenges, received support or recognition for doing so effectively, or seen others model such competence. Such experience may not necessarily be developed simply with years of experience as a GSA advisor. Without deliberate reflection or exposure to multiple forms of oppression or their intersection in practice, advisors may not develop the skills or confidence needed to effectively support youth navigating overlapping forms of oppression related to race, ethnicity, immigration, and their intersection with oppression related to sexuality and gender diversity. Some advisors with less experience may have recently completed training that embeds intersectionality more centrally, giving them a stronger foundation in this area. Moreover, advisors' racialized lived experiences (e.g., experiences of marginalization or systemic exclusion) may serve as a foundation for developing perspective‐taking and confidence in navigating complex identity‐related issues and contexts (e.g., Poteat & Scheer, 2016; Spanierman & Smith, 2017).

Contextual features may operate in a similar way in contributing to advisors' REISE: Advisors serving GSAs with more members of color may have more opportunities to engage with race‐ and ethnicity‐related issues, making experiential growth in REISE more likely (Malinen et al., 2013). Such growth may be further strengthened by a supportive school context that facilitates culturally responsive curricula and race‐ or ethnicity‐focused events, and a GSA with an inclusive and affirming climate. Building on evidence that inclusive school climates and policies support GSA advisors' functioning (Watson et al., 2010), our findings add that such contexts are uniquely linked to advisors' confidence in navigating racial‐ethnic, immigration, and intersectional issues in their advising practice (i.e., REISE). These environments likely offer repeated opportunities to engage with intersectional issues in practice, as dialogue, programming, and daily interactions can provide advisors with the conditions needed to build confidence through learning, feedback, and reflection (Bandura, 1997). Alternatively, given the cross‐sectional findings, it is plausible that advisors with high REISE contribute to creating more inclusive climates or attracting diverse members. Equally, GSAs with a racially diverse membership may seek out or retain advisors who already demonstrate competence in addressing this constellation of concerns. These potentially reciprocal processes underscore the need for longitudinal research that can disentangle the bidirectional dynamics between advisor characteristics and the social ecology of GSAs.

Lastly, these findings underscore the importance of distinguishing between general self‐efficacy and specific forms of efficacy tied to intersections and lived experiences, like REISE. As advisors of color reported higher REISE than White advisors even after accounting for gender identity and sexual orientation, racial identity may relate uniquely to intersectional competence. REISE likely captures a distinct construct that cannot be fully explained by generalized confidence or experience alone, reinforcing the value of examining identity‐specific and intersectional efficacy beliefs when studying advisor roles in GSAs and other equity‐focused educational contexts.

### Implications of advisors' REISE for youth members' socioemotional experiences

The second research question focused on the associations between advisors' REISE and youth members' socioemotional experiences throughout the academic year. At the interpersonal level, students in GSAs led by advisors with higher REISE perceived both the GSA in general as more supportive and perceived their advisors as more responsive to their emotional, social, and identity‐related needs. Extending findings from general school contexts showing that teachers' culturally relevant practices are associated with safer school climates (Bayram Özdemir et al., 2024; Byrd, 2016), our results indicate that this pattern extends to GSAs, where advisors' REISE may be associated with a more supportive affective climate within the group. Advisors with greater REISE may also be more likely to foster, or be embedded in, GSA environments in which students can openly share experiences, access emotional and social support, and perceive their advisor as sensitive and responsive, consistent with the PYD framework (Lerner et al., 2015).

Beyond the GSA, advisors' REISE was associated with members' reduced experiences of bullying‐victimization and increased positive affect over the course of the school year. This pattern suggests that the associations between REISE and youth well‐being may extend beyond the immediate GSA context. Repeated interactions with advisors who are attuned to youths' intersectional experiences may be associated with youths' emotional security, coping capacities, and willingness to seek support when facing identity‐related stressors. Consistent with Bandura's (1997) social cognitive theory, such experiences may gradually support youths' perceived ability to navigate stigma and discrimination across contexts, which may help explain associations with lower victimization experiences and more sustained positive affect over time. These findings extend prior work showing that GSAs are associated with lower levels of bullying‐victimization, particularly among students of color (Van Vliet et al., 2025), by suggesting, that advisors—through REISE—may represent a potentially important yet previously underexamined resource associated with these protective patterns.

The prospective associations between advisors' REISE and youths' broader socioemotional experiences may also partly reflect the broader school contexts in which these advisors operate. However, REISE explained these experiences beyond baseline indicators of school inclusivity (i.e., the schools' initial culturally inclusive curriculum and events). At the same time, advisors with higher REISE may actively contribute to shaping more affirming school climates over time, for example by advocating for inclusive practices, organizing schoolwide events, or fostering affirming norms within the broader school community (Day et al., 2019; Poteat & Day, 2025). In this way, broader school climate processes may partly help explain why prospective associations emerged primarily for youths' broader socioemotional experiences outside the immediate GSA context. Moreover, advisors with higher REISE may also be more likely to work within schools characterized by other unmeasured aspects of affirming school climate, such as more culturally inclusive attitudes among staff, which may also contribute to these longitudinal associations. Altogether, potential developmental and ecological processes that accumulate over time and extend beyond the immediate GSA context may help explain why prospective associations were primarily observed for victimization and positive affect. GSA‐specific experiences may be more proximal and context‐dependent, reflecting youths' immediate interactions within the group and therefore fluctuating over time with group dynamics and situational factors.

### REISE as a broadly beneficial relational resource

Observed associations between advisors' REISE and youth members' experiences appeared consistent across student subgroups based on race or victimization levels. The absence of moderation effects suggests that advisor intersectional efficacy may operate through broader climate‐level mechanisms, consistent with ecological systems theory, whereby changes at the relational or group level relate to the experiences of all youth within the setting. REISE may serve as a broadly relevant relational resource within GSAs, rather than one whose relevance is confined to students occupying more marginalized social positions. It may reflect a general capacity to foster inclusive and affirming group environments that are experienced positively by a broad range of students, aligning with literature emphasizing that inclusive practices and policies benefit all students (e.g., Russell et al., 2021). Advisors who report greater confidence in addressing race‐ and ethnicity‐related concerns and concerns faced among immigrant youth may be more likely to engage in practices or establish norms that promote belonging, recognize dynamics of inclusion and exclusion as they arise, and respond in ways aligned with equity and affirmation.

### Limitations

While the findings contribute novel insights into the role of racial‐ethnic, immigration, and intersectional self‐efficacy within GSA contexts, several limitations should be considered when interpreting the results. First, REISE was assessed at a single time point. We conceptualized REISE as a relatively stable, trait‐like construct, as it reflects advisors' underlying REISE rather than momentary fluctuations. In the absence of targeted interventions, such self‐efficacy is unlikely to change substantially over the relatively short two‐month time interval between waves. Nevertheless, longitudinal designs that capture REISE at multiple time points would provide greater clarity about developmental patterns and directionality.

Second, although the study examined several advisor characteristics, it did not include some potentially relevant variables, such as advisors' prior training in equity, diversity, and inclusion or their personal experiences with discrimination or marginalization. Such factors may contribute to the development of REISE and may interact with contextual features in complex ways. Future research could explore these additional dimensions to better understand the conditions under which REISE emerges and how it may be strengthened.

In terms of scope, the present study focused specifically on race‐ and ethnicity‐related self‐efficacy in general and when intersecting with 2SLGBTQ+ identities. However, other axes of identity—such as disability, religion, or class background—as well as distinctions within 2SLGBTQ+ identities (e.g., sexual orientation versus gender identity) may shape youth experiences in different ways and require more differentiated or domain‐specific forms of efficacy. In line with this, although the advisors' self‐efficacy subscale items were aligned with key advisor roles in GSAs and with roles described in Rhodes' (2005) mentorship model, and correlations between subscales supported their distinctiveness and theoretical coherence, our sample size precluded more extensive evaluation of the measures. Specifically, we were unable to formally test the distinctiveness of the different self‐efficacy dimensions or the potential presence of a higher‐order efficacy factor. Future research would benefit from further developing and validating measures for GSA advisors, as part of a broader effort to better capture their contributions within GSAs and address their needs in this role.

Further, responses were collected confidentially and score distributions did not indicate strong ceiling effects, suggesting limited socially desirable responding. However, this cannot be fully ruled out, and future research could further address this by emphasizing honest responding in instructions or including dedicated social desirability measures. Lastly, the findings cannot be generalized beyond the sampled school contexts, given the important role of contextual embeddedness of GSAs in the studied associations.

Altogether, despite the challenges of collecting data within GSAs and among marginalized school staff and student populations in the current sociopolitical climate, our findings highlight the value and necessity of longitudinal research to capture these processes.

## CONCLUSIONS

In summary, the present study highlights GSA advisors' REISE as a key relational resource within GSAs. Rather than reflecting time‐based experience (e.g., age, years of advising), REISE appears to be linked to advisors' identities, perceived competence, and the broader school and group contexts in which they work. Importantly, higher REISE coincides with more supportive and affirming experiences for youth members across the school year, irrespective of the school's inclusive curriculum or events. These patterns suggest that advisors' capacity to engage with racial, ethnic, immigration and 2SLGBTQ+ intersectional dynamics may shape GSA climates in ways that benefit students broadly, regardless of background. By integrating ecological, social cognitive, and PYD perspectives, and leveraging multi‐informant, longitudinal data, the findings underscore the central role of REISE in explaining youth outcomes and GSA experiences.

Future research should build on this foundation by identifying the specific advisor behaviors, interactions, and support mechanisms that underlie higher REISE, with the goal of informing professional development and advisor training. Using larger sample sizes, such research could explore heterogeneous associations across student subgroups with different experiences, and whether a more global measure of intersectional self‐efficacy encompassing multiple identity domains could offer added explanatory value. Additionally, future studies might investigate potential mediating processes, such as youth members' emotion regulation, feelings of empowerment, coping strategies, or perceptions of safety and belonging, as well as school‐level processes such as inclusive norms potentially fostered by advisors. A more granular understanding of how advisor REISE may relate to these intermediate psychological processes could help clarify the pathways through which supportive environments are fostered.

From a practical perspective, the findings point to the importance of professional development approaches that prioritize building advisors' perceived capability around race and identity through reflective practice, peer learning, and exposure to intersectional perspectives, including training led by advisors or practitioners with lived experience of marginalization. They also underscore the need for targeted support for advisors in less diverse school contexts and for efforts to diversify the pool of GSA advisors, particularly in predominantly white school systems. Finally, the association between higher REISE and school‐level supports such as culturally responsive curricula, identity‐affirming events, and inclusive climates highlights the role of structural investments in creating conditions that strengthen advisors' confidence and capacity. Together, these findings position advisors' REISE as a promising and actionable focus for strengthening intersectional competence in GSA practice, training, and research.

## AUTHOR CONTRIBUTIONS

**Alex Mascarino:** Conceptualization; writing – original draft; writing – review and editing; validation; visualization; methodology; investigation. **Robert A. Marx:** Conceptualization; investigation; writing – original draft; writing – review and editing; methodology; funding acquisition. **Wouter J. Kiekens:** Conceptualization; writing – original draft; writing – review and editing; methodology; investigation. **Tessa M. L. Kaufman:** Conceptualization; investigation; writing – original draft; writing – review and editing; methodology; validation; visualization; formal analysis. **Maria Di Stasio:** Conceptualization; investigation; writing – original draft; writing – review and editing; methodology. **V. Paul Poteat:** Conceptualization; investigation; funding acquisition; writing – original draft; methodology; writing – review and editing.

## FUNDING INFORMATION

This work was supported by Institute of Education Sciences, Grant R305A190165.

## CONFLICT OF INTEREST STATEMENT

The authors have no conflict of interest to declare.

## ETHICS STATEMENT

All procedures were in accordance with the ethical standards of the institutional research committee and with the 1964 Helsinki declaration and its later amendments or comparable ethical standards. All study procedures were approved by Boston College IRB, protocol 19.280.01.

## CONSENT STATEMENT

Informed assent was obtained from all youth participants included in the study. A waiver of parental consent was obtained from the IRB for youth participating in some schools while adult consent was obtained from GSA adult advisors; parental consent was obtained for youth participating in school districts with this requirement.

## Supporting information


**Supplementary Material A.** Self‐efficacy scales: Full item set.
**Table A1**. Items for advisors' REISE.
**Table A2**. Items for advisors' general self‐efficacy.
**Table A3**. Items for advisors' self‐efficacy related to gender diversity.
**Table A4**. Items for advisors' self‐efficacy related to sexual orientation.
**Supplementary Material B**. Correlations between advisors' REISE and youth member experiences.
**Table B1**. Correlations between REISE and student outcomes across waves.

## Data Availability

The data that support the findings of this study are available on request from the corresponding author. The data are not publicly available due to privacy or ethical restrictions.
