# Supplementary material for: How racial‐ethnic, immigration, and 2SLGBTQ+ intersectional self‐efficacy of gender‐sexuality alliance advisors shapes youth members' positive experiences
Source: J Res Adolesc. 2026 Aug 3;36(3):e70243. doi: 10.1111/jora.70243 (PMC13430430; doi:10.1111/jora.70243)
Supplement: Supplementary file 1 — Supplementary Material A. Self‐efficacy scales: Full item set. Table A1. Items for advisors' REISE. Table A2. Items for advisors' general self‐efficacy. Table A3. Items for advisors' self‐efficacy related to gender diversity. Table A4. Items for advisors' self‐efficacy related to sexual orientation. Supplementary Material B. Correlations between advisors' REISE and youth member experiences. Table B1. Correlations between REISE and student outcomes across waves. [file JORA-36-0-s001.docx]

**Supplementary Material A**

**Self-Efficacy Scales: Full Item Set**

**Advisors’ REISE**

**Table A1***Items for Advisors’ REISE.*

| How competent do you currently feel to do the following? | Not at all | A little | Moderately | Quite a bit | Extremely |
| --- | --- | --- | --- | --- | --- |
| 1. Talk about unique experiences that LGBTQ students of color face. | 1 | 2 | 3 | 4 | 5 |
| 1. Address issues and topics related to the intersection of race and sexual orientation or gender identity. | 1 | 2 | 3 | 4 | 5 |
| 1. Take actions to address instances of racism that students of color face. | 1 | 2 | 3 | 4 | 5 |
| 1. Support students of color on issues related to race and ethnicity | 1 | 2 | 3 | 4 | 5 |
| 1. Talk about issues and topics related to immigration or experiences of immigrant youth. | 1 | 2 | 3 | 4 | 5 |
| 1. Talk about students’ experiences in different cultures. | 1 | 2 | 3 | 4 | 5 |
| 1. Advocate or speak up on behalf of students of color to other students at the school. | 1 | 2 | 3 | 4 | 5 |
| 1. Advocate or speak up on behalf of students of color to other teachers or administrators at the school. | 1 | 2 | 3 | 4 | 5 |

**General Self-Efficacy**

**Table A2**

*Items for Advisors’ General Self-Efficacy.*

| How competent do you currently feel to do the following? | Not at all | A little | Moderately | Quite a bit | Extremely |
| --- | --- | --- | --- | --- | --- |
| 1. Support students in the GSA who are struggling academically. | 1 | 2 | 3 | 4 | 5 |
| 1. Work with students in the GSA on issues related to their family. | 1 | 2 | 3 | 4 | 5 |
| 1. Work with students in the GSA who have not been coming to school or who have not been going to certain classes. | 1 | 2 | 3 | 4 | 5 |
| 1. Provide support to a student who is experiencing bullying or discrimination at school. | 1 | 2 | 3 | 4 | 5 |

**2SLGBTQ+ related Self-Efficacy**

**Table A3**

*Items for Advisors’ Self-Efficacy related to Gender Diversity.*

| How competent do you currently feel to do the following? | Not at all | A little | Moderately | Quite a bit | Extremely |
| --- | --- | --- | --- | --- | --- |
| 1. Talk about unique experiences that transgender and non-binary students face. | 1 | 2 | 3 | 4 | 5 |
| 1. Address issues and topics related to gender identity and expression. | 1 | 2 | 3 | 4 | 5 |
| 1. Explain the difference between gender identity and sexual orientation. | 1 | 2 | 3 | 4 | 5 |
| 1. Support transgender and non-binary students on issues related to gender identity and expression. | 1 | 2 | 3 | 4 | 5 |
| 1. Take actions to address instances of bullying or discrimination that transgender and non-binary students face. | 1 | 2 | 3 | 4 | 5 |
| 1. Advocate or speak up on behalf of transgender and non-binary students to other students at the school. | 1 | 2 | 3 | 4 | 5 |
| 1. Advocate or speak up on behalf of transgender and non-binary students to other teachers or administrators at the school. | 1 | 2 | 3 | 4 | 5 |

**Table A4**

*Items for Advisors’ Self-Efficacy related to Sexual Orientation.*

| How competent do you currently feel to do the following? | Not at all | A little | Moderately | Quite a bit | Extremely |
| --- | --- | --- | --- | --- | --- |
| 1. Talk about unique experiences that LGBQ students face. | 1 | 2 | 3 | 4 | 5 |
| 1. Address issues and topics related to sexual orientation. | 1 | 2 | 3 | 4 | 5 |
| 1. Take actions to address instances of bullying or discrimination that LGBQ students face. | 1 | 2 | 3 | 4 | 5 |
| 1. Talk about students’ experiences from different sexual orientation identities. | 1 | 2 | 3 | 4 | 5 |
| 1. Support LGBQ students on issues related to sexual orientation. | 1 | 2 | 3 | 4 | 5 |
| 1. Advocate or speak up on behalf of LGBQ students to other students at the school. | 1 | 2 | 3 | 4 | 5 |
| 1. Advocate or speak up on behalf of LGBQ students to other teachers or administrators at the school | 1 | 2 | 3 | 4 | 5 |

**Supplementary Material B**

**Correlations between advisors’ REISE and youth member experiences**

Cross-sectional associations between and within REISE and member experiences that were corrected for nesting at the GSA-level (Table B1) ranged from small to strong in magnitude at T1, with several moderate to large correlations, particularly between REISE, perceived advisor responsiveness, and positive affect, and a strong negative association with victimization. At T2 and T3, correlations were generally smaller in magnitude and more variable, with few to no statistically significant associations. This pattern likely reflects both a weakening of cross-sectional alignment over time and reduced statistical power at later waves due to smaller sample sizes. In contrast, stability correlations within constructs were generally moderate to strong across waves, particularly for perceived advisor responsiveness and advocacy engagement, indicating relatively consistent individual differences over time. Overall, these findings suggest that while the constructs themselves show temporal stability, their interrelations are less stable and may be sensitive to sample size and measurement timing.

**Table B1**

*Correlations between REISE and Student Outcomes across Waves.*

| Variable | 1 | 2 | 3 | 4 | 5 | 6 |
| --- | --- | --- | --- | --- | --- | --- |
| 1. REISE | — |  |  |  |  |  |
| **GSA-related** |  |  |  |  |  |  |
| 2. GSA’s social support | 0.52 / 0.05 / 0.41 | 0.73 / 0.38 / 0.76 |  |  |  |  |
| 3. Perceived advisors’ responsiveness | 0.59** / -0.04 / 0.38 | 0.82 / 0.94 / 0.98*** | 0.75 / 0.71 / 0.84 |  |  |  |
| 4. Advocacy engagement | 0.29 / 0.44 / 0.49 | 0.89 / 0.60 / -0.04 | 0.71 / 0.70 / -0.03 | 0.87 / 0.52 / 0.67 |  |  |
| **General** |  |  |  |  |  |  |
| 5. Victimization | -0.50* / -0.29 / 0.21 | -0.17 / -0.49 / -0.11 | -0.66 / -0.32 / -0.19 | -0.06 / 0.00 / -0.24 | 0.82* / 0.64 / 0.87 |  |
| 6. Positive affect | 0.65*** / 0.40 / -0.01 | 0.26 / 0.76 / 0.29 | 0.63 / 0.71* / 0.24 | 0.26 / 0.68 / -0.37 | -0.74*** / -0.65 / 0.78 | 0.81 / 0.53 / 0.32 |

*Note*. Values represent correlations at T1/T2/T3. Diagonal elements represent stability correlations across waves (T1–T2 / T1–T3 / T2–T3). * p < .05, ** p < .01, *** p < .001.
